# Key components of chemotherapy for thymic malignancies: a systematic review and pooled analysis for anthracycline-, carboplatin- or cisplatin-based chemotherapy

**DOI:** 10.1007/s00432-014-1800-6

**Published:** 2014-08-22

**Authors:** Yusuke Okuma, Makoto Saito, Yukio Hosomi, Toshikazu Sakuyama, Tatsuru Okamura

**Affiliations:** 1 Department of Thoracic Oncology and Respiratory Medicine, Tokyo Metropolitan Cancer and Infectious diseases Center Komagome Hospital, 3-18-22 Honkomagome, Bunkyo, Tokyo, 113-8677 Japan; 2 Division of Clinical Research Support, Tokyo Metropolitan Cancer and Infectious diseases Center Komagome Hospital, Tokyo, Japan; 3 Division of Oncology, Research Center for Medical Sciences, The Jikei University School of Medicine, Tokyo, Japan

**Keywords:** Thymic malignancies, Thymic carcinoma, Thymoma, Rare cancer, Chemotherapy

## Abstract

**Purpose:**

Thymic malignancies, comprising thymoma and thymic carcinoma, are rare. Consequently, optimal chemotherapy for advanced thymic malignancies remains controversial. Platinum-based chemotherapy is currently the consensus treatment based on the results of single-arm phase II trials and retrospective investigations. However, comparison of cisplatin-based and carboplatin-based chemotherapy has yet to be undertaken; the effectiveness of the addition of anthracycline also remains uncertain.

**Methods:**

In the present study, clinical trials and retrospective data regarding platinum-based chemotherapy were analyzed. The endpoint was the response rate to each chemotherapy. For advanced thymoma, we compared platinum with anthracycline-based chemotherapy and platinum with non-anthracycline-based chemotherapy. For advanced thymic carcinoma, anthracycline-based versus non-anthracycline-based chemotherapy and carboplatin-based versus cisplatin-based chemotherapy were compared. This analysis included a retrospective study of response of advanced thymic carcinoma to irinotecan and cisplatin in our institution.

**Results:**

The response rate for the 314 patients from 15 studies with advanced thymoma, including both prospective and retrospective data, was 69.4 % [95 % confidence interval (CI) 63.1–75.0 %] for platinum with anthracycline-based chemotherapy and 37.8 % (95 % CI 28.1–48.6 %; *p* < 0.0001) for platinum with non-anthracycline-based chemotherapy. The response rates after anthracycline-based and non-anthracycline-based chemotherapy for advanced thymic carcinoma were similar (41.8 vs. 40.9 %; *p* < 0.91), whereas the response rates after cisplatin-based and carboplatin-based chemotherapy for advanced thymic carcinoma differed significantly (53.6 vs. 32.8 %; *p* *=* 0.0029) in 206 patients from 10 studies.

**Conclusions:**

Platinum with anthracycline-based chemotherapy is an optimal combination for advanced thymoma. For advanced thymic carcinoma, cisplatin-based chemotherapy may be superior to carboplatin-based chemotherapy.

## Introduction

Standard chemotherapy for thymomas and thymic carcinomas, collectively called thymic malignancies, remains undefined because of their rarity. For common cancers, standard chemotherapy is determined by means of phase III trials that compare experimental chemotherapy with standard chemotherapy. As is common with rare cancers, evaluation of chemotherapy for thymomas and thymic carcinomas is limited to single-arm phase II trials or retrospective analyses involving small numbers of patients. Therefore, the primary endpoint for clinical trials regarding thymoma and thymic carcinoma is usually response rate and not time to event.

The pathological classification of such tumors has been confused because of the transition to modern classifications. At present, the 2004 World Health Organization (WHO) classification (Travis et al. 2004) based on clinical prognosis has been agreed by consensus (Huang et al. 2010). Furthermore, thymoma is a functional immunological tumor that retains the immunological characteristics of the thymus, whereas thymic carcinoma is associated with loss of organotypic status. Thymoma and thymic carcinoma are rare cancers with an annual incidence of 1.3 to 3.2/100,000 persons/year (de Jong et al. 2008; Engel et al. 1999; Engels 2010). Based on the definition of the RARECARE project supported by the European Commission, rare cancer has an annual incidence of <6/100,000 persons/year. Standard chemotherapy for advanced thymoma and thymic carcinoma remains controversial because of the limited amount of available therapeutic evidence. However, there is a high level of consensus that the key drugs for the treatment of thymoma are anthracycline and cisplatin (Schmitt and Loehrer 2010), and the chemotherapy administered for thymic carcinoma is generally based on the chemotherapy administered for thymoma. Furthermore, the diagnosis of thymic malignancies is based on modern criteria reported by Bernatz et al. (1961), the Levine and Rosai classification (Levine and Rosai 1978), the Müller-Hermelink classification (1986), the 1999 WHO classification (Rosai 1999) or the 2004 WHO classification (Travis et al. 2004). Recently, prospective and retrospective investigations of chemotherapy for thymic carcinoma have been increasing, but well-designed studies are uncommon and the diagnostic reliability regarding thymic carcinoma is not always high (Weksler et al. 2013; Zucali et al. 2013).

Consequently, we conducted a pooled analysis to extract and compile the data from published reports and to clarify the key drugs used for the treatment of advanced thymoma and thymic carcinoma. Furthermore, our previously published retrospective data regarding the clinical outcome of cisplatin and irinotecan combination chemotherapy for advanced thymic carcinoma have been updated (Okuma et al. 2011).

## Methods

### Selection criteria for literature and our updated data

A systematic search of the PubMed databases was performed to identify all prospective clinical trials and retrospective studies involving first-line platinum-based chemotherapy for advanced or recurrent thymomas and thymic carcinomas. The search included articles from 1990 to 2013 using the search string “thymoma” OR “thymic carcinoma” AND “chemotherapy.” Among these retrieved articles, non-English language articles, case reports and reviews were excluded. The abstracts of articles of potential relevance were reviewed, and articles that were clearly relevant were selected for further analysis. Additionally, studies presented at the 2013 and 2014 ASCO annual meetings were searched to ensure that the most up-to-date articles were included in the analysis. Unfortunately, clinical trials for thymoma included patients with thymic carcinoma because of the prior criteria for pathological diagnosis. The study included all published reports that contained clinical outcomes of platinum-based combination chemotherapy (cisplatin-based vs. carboplatin-based chemotherapy and anthracycline vs. non-anthracycline combination chemotherapy) for advanced or recurrent thymoma and thymic carcinoma, with some including induction chemotherapy. In the studies regarding induction chemotherapy followed by surgery or radiotherapy, a small number of patients with Masaoka Stage III were included. In such cases, the response rate to chemotherapy alone was reported, and the time to events was ignored.

We have previously published the outcomes of nine cases of cisplatin and irinotecan combination chemotherapy (Okuma et al. 2011); in addition, we retrospectively assessed 12 such cases up until 2013 in the present study and combined the data with the above published data, including the response rates. A retrospective review was performed to collect data on the outcomes of 12 consecutive patients treated with cisplatin and irinotecan chemotherapy for advanced thymic carcinoma at Masaoka-Koga stage IVa, IVb or recurrent disease. Thymic carcinoma was confirmed by hematoxylin and eosin staining and immunohistochemistry using CD5 and/or CD117 (c-KIT) to exclude other malignant thoracic tumors at the time of initial diagnosis. The pathological review was consistently performed by a specialist in thymic malignancies (T Hishima). Recurrent disease was defined as disease that was not responsive to treatment with curative intent; all patients with recurrent disease were chemotherapy naïve and underwent chemotherapy with palliative intent. Recurrent disease was determined using chest computed tomography, magnetic resonance imaging, positron emission tomography, or bone scanning. Histology was also classified according to the 2004 WHO classification, and staging was determined using the Masaoka-Koga staging system (2010). Data were collected in accordance with the International Thymic Malignancy Interest Group (ITMIG) standard definitions and policies (Girard et al. 2011). The medical records and laboratory data for each patient were retrieved for analysis, and treatments for thymic carcinoma were assessed. Progression-free survival (PFS) was defined as the time from the first cycle of chemotherapy to the first clinical evidence of progressive disease, early discontinuation of treatment or death from any cause. Overall survival (OS) was defined as the time from the first cycle of treatment to the time of death from any cause or the last follow-up. Because of the retrospective nature of the data, these end points were chosen to reflect clinical practice. Assessment of response to chemotherapy was achieved using the Response Evaluation Criteria in Solid Tumors criteria version 1.1 (RECIST 1.1).

### Patient selection and statistics

The criteria for the selection included in these published reports were patients with cytologically or histologically proven advanced or recurrent thymoma or thymic carcinoma, diagnosed using the modern histological classifications. Treatment schedule, response, survival assessment and statistical analyses were performed to the extent that was possible. In all of the identified reports, patients with advanced or recurrent thymic malignancies underwent platinum-based combination chemotherapy with anthracyclines, carboplatin or cisplatin. The treatment response was determined using either RECIST criteria version 1.0, version 1.1., the WHO criteria, or the European Cooperative Oncology Group (ECOG) criteria. The diagnosis was based on modern classifications. Time to event (OS or PFS) was used as an endpoint in the present study as in the published literature; however, the period from the initiation of treatment to the date when disease progression or death was observed was used.

The proportion of patients having advanced recurrent thymomas or thymic carcinomas, including neuroendocrine carcinoma, and the response rates were compared using the chi-square test. Statistical significance was defined as *p* < 0.05. Statistical analyses were performed using the JMP11 software program (SAS Institute Inc., Cary, NC, USA).

The potential for publication bias of the response rates in reported studies was assessed using funnel plots, with appropriate accuracy intervals.

## Results

The process of identifying studies eligible for inclusion in our analysis was as follows: first, we reviewed 57 full articles from 393 published studies and meeting abstracts related to thymomas and excluded 208 case reports and review articles. Of the total 160 published articles and meeting abstracts on thymic carcinoma, we reviewed 78 full articles and excluded 82 case reports and review articles. Of these, 15 studies met the inclusion criteria.

### Characteristics of the selected studies

The preliminary analysis encompassed 15 studies involving a total of 314 patients with advanced or recurrent thymoma who were treated using platinum with or without anthracycline chemotherapy. In these studies, thymic carcinoma was included because it was considered as being type C thymoma. The studies included ten prospective studies and five retrospective studies (Table 1). In addition, a total of 206 patients with advanced thymic carcinoma were included in a pooled analysis of platinum with or without anthracycline chemotherapy in ten studies, consisting of four prospective studies and six retrospective studies (Tables 2, 3).

**Table 1:** Unified response rates of advanced thymoma patients treated with anthracycline-based or non-anthracycline-based chemotherapy regimens

Regimen	Author, year	Study design	Stage	No. of patients	Responders	RR	PFS	OS
*Anthracycline*-*containing regimens*								
ADOC	Fornasiero et al. (1991)	S	III/IVa/IVb	37	34	91.8 %	12 mos	15 mos
PAC	Loehrer et al. (1994)	G	IV	29	15	51.7 %	11.8 mos	37.7 mos
PAC (+XRT)	Loehrer et al. (1997)	G	III	23	16	69.6 %	–	93 mos
ADOC	Rea et al. (1993)	S	III/IVa	16	12	75.0 %	–	66 mos
ADOC	Berruti et al. (1999)	S	III/IVa	16	13	81.3 %	33.2 mos	47.5 mos
PAC	Kim et al. (2004)	G	III/IVa/IVb	22	17	77.3 %	–	–
PAE (+XRT)	Lucchi et al. (2006)	S	III/IVA	30	22	73.3 %	–	–
CAMP	Yokoi et al. (2007)	S	IVa/IVb	14	13	92.9 %	–	–
Dose-dense CODE	Kunitoh et al. (2009)	G	IVa/IVb	27	16	59.3 %	0.79 year	6.1 year
CarboAMR	Kawashima et al. (2013)	G	Invasive	18	3	16.7 %	7.6 mos	Not reached
Total				232	161	69.4 %		
*Non*-*anthracycline*-*containing regimens*								
PE	Giaccone et al. (1996)	G	III/IV/rec	16	9	56 %	2.2 year	4.3 year
VIP	Loehrer et al. (2001)	G	III/IVa/IVb	20	7	35 %	11.9 mos	31.6 mos
VIP	Grassin et al. (2011)	G	IIIB/IVA/IVB	16^a^	4^a^	25 %^a^	13.1 mos	Not reached
CarboPTX	Takeda et al. (2013)	G	III/IVa/IVb	21	6	42.9 %	16.7 mos	Not reached
CDDP/DTX	Park et al. (2013)	G	III/IVa/IVb	9	5	55.6 %	–	–
Total				82	31	37.8 %		

*G* prospective multicenter group phase II trial,* S* single-center experience,* mos* months,* RR* objective response rate,* ADOC* adriamycin, cisplatin, vincristine and cyclophosphamide,* PAC* cisplatin, adriamycin and cyclophosphamide,* PAE* cisplatin, adriamycin and etoposide,* CAMP* PAC = cisplatin, adriamycin, methylprednisolone and cyclophosphamide,* CODE* adriamycin, cisplatin, vincristine and etoposide,* PE* cisplatin and etoposide,* VIP* vincristine, ifosfamide and cisplatin,* CarboPTX* carboplatin and paclitaxel

^a^Including four patients with thymic carcinoma in the VIP trial

**Table 2:** Unified response rates of advanced thymic carcinoma patients treated with anthracycline-based or non-anthracycline-based chemotherapy regimens

Regimen	Author, year	Study design	Stage	No. of patients	Responders	RR	PFS (mos)	MST (mos)
*Anthracycline*-*based chemotherapy*								
ADOC	Agatsuma et al. (2011)	S	IVa/IVb	34	17	50 %	N/A	21.3
CODE	Yoh et al. (2003)	S	III/IVa/IVb	12	5	42 %	5.6	46
CarboAMR^a^	Kawashima et al. (2013)	G	Invasive^a^	33	11	30 %	7.6	27.3
Total				79	33	41.8 %		
*Non*-*anthracycline*-*based chemotherapy*								
VIP	Grassin et al. (2011)	S	III/IVa/IVb	8	2	25 %	–	–
CarboPTX	Lemma et al. (2011)	G	III/IVa/IVb	23	5	21.7 %	5.0	20.0
CarboPTX	Igawa et al. (2010)	S	IVa/IVb	11	4	36 %	7.9	22.7
CarboPTX	Takeda et al. (2013)	G	III/IVa/IVb	39	14	35.9 %	7.52	Not reached
CarboPTX	Furugen et al. (2011)	S	IVa/IVb/rec	16	6	37.5 %	8.6	49.4
CDDP/DTX^b^	Park et al. (2013)	G	III/IVa	18	12	66.7 %	–	–
CDDP/CPT-11	Present study	S	IVa/IVb/rec	12	9	75.0 %	7.4	52.4
Total				127	52	40.9 %		

*G* prospective multicenter group phase II trial,* S* single-center experience,* RR* response rate,* PFS* progression-free survival,* OS* overall survival,* ADOC* adriamycin, cisplatin, vincristine and cyclophosphamide,* CODE* adriamycin, cisplatin, vincristine and etoposide,* CarboAMR* carboplatin and amrubicin,* CarboPTX* carboplatin and paclitaxel,* CDDP/CPT-11* cisplatin and irinotecan,* CDDP/DTX* cisplatin/docetaxel

^a^The subset for thymic carcinoma

^b^The accrual criteria were defined as “invasive” thymoma or thymic carcinoma, not according to any defined staging system. The chemotherapeutic setting was within the two previous regimens

**Table 3:** Unified response rates of advanced thymic carcinoma patients treated with cisplatin-based or carboplatin-based chemotherapy regimens

Regimen	Author, year	Study design	Stage	No. of patients	Responders	RR	PFS (mos)	MST (mos)
*Cisplatin*-*based chemotherapy*								
ADOC	Agatsuma et al. (2011)	S	IVa/IVb	34	17	50 %	N/A	21.3
CODE	Yoh et al. (2003)	S	III/IVa/IVb	12	5	42 %	5.6	46
VIP	Grassin et al. (2011)	S	III/IVa/IVb	8	2	25 %	–	–
CDDP/DTX^a^	Park et al. (2013)	G	III/IVa	18	12	66.7 %	–	–
CDDP/CPT-11	Present study	S	IVa/IVb/rec	12	9	75.0 %	7.4	52.4
Total				84	45	53.6 %		
*Carboplatin-based chemotherapy*								
CarboPTX	Lemma et al. (2011)	G	III/IVa/IVb	23	5	21.7 %	5.0	20.0
CarboPTX	Igawa et al. (2010)	S	IVa/IVb	11	4	36 %	7.9	22.7
CarboPTX	Takeda et al. (2013)	G	III/IVa/IVb	39	14	35.9 %	7.52	Not reached
CarboPTX	Furugen et al. (2011)	S	IVa/IVb/rec	16	6	37.5 %	8.6	49.4
CarboAMR^a^	Kawashima et al. (2013)	G	Invasive^a^	33	11	30 %	7.6	27.3
Total				122	40	32.8 %		

*G* prospective multicenter group phase II trial,* S* single-center experience,* RR* response rate,* PFS* progression-free survival,* OS* overall survival,* ADOC* adriamycin, cisplatin, vincristine and cyclophosphamide,* CODE* adriamycin, cisplatin, vincristine and etoposide,* CarboAMR* carboplatin and amrubicin,* CarboPTX* carboplatin and paclitaxel,* CDDP/CPT-11* cisplatin and irinotecan,* CDDP/DTX* cisplatin/docetaxel

^a^The subset for thymic carcinoma

A total of 12 patients who were treated with cisplatin and irinotecan combination chemotherapy as palliative-intent chemotherapy were also evaluated. Nine partial responses (75.0 %) and two stable diseases [16.7 %; total disease control was observed in 11 patients (91.7 %)] were recorded. One patient had progressive disease. There were no complete responders. The median PFS was 7.4 months [95 % confidence interval (CI) 2.2–9.2 months), while the median OS was 52.4 months (95 % CI 9.4–114.2 months). The 1- and 2-year survival rates based on the Kaplan–Meier analysis were 88.9 and 66.7 %, respectively.

### Response to platinum-based chemotherapy in advanced thymoma and thymic carcinoma

The response rate of thymoma to anthracycline-based chemotherapy was 69.4 % (95 % CI 63.1–75.0 %) and 37.8 % (95 % CI 28.1–48.6 %) to non-anthracycline-based chemotherapy. The difference in the response rates between anthracycline-based chemotherapy and non-anthracycline-based chemotherapy with cisplatin was statistically significant (*χ* ^2^ test; *p* < 0.0001). The response rates of thymic carcinoma to anthracycline-based chemotherapy were 41.8 % (95 % CI 31.5–52.8 %) and 40.9 % (95 % CI 32.8–49.6 %) to non-anthracycline-based chemotherapy (Table 2); there was no significant difference in the response rates (*χ* ^2^ test; *p* < 0.82). The response rates of thymic carcinoma were 53.6 % (95 % CI 43.0–63.8 %) to cisplatin-based chemotherapy and 32.8 % (95 % CI 25.1–41.5 %) to carboplatin-based chemotherapy (Table 3); the difference in the response rates was significant (*χ* ^2^ test; *p* = 0.0029).

### Publication bias

**Fig. 1:**
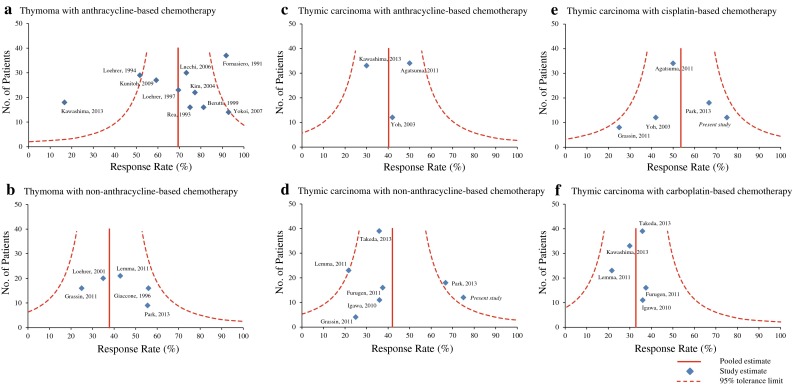
Funnel plots using response rates as an outcome for **a**, **b** thymoma and **c**–**f** thymic carcinoma in each chemotherapy category (anthracycline-based vs. non-anthracycline-based and cisplatin-based vs. carboplatin-based)

Potential publication bias was assessed using funnel plots with response rates as the outcome. The funnel plots were basically symmetrical for each of the regimen categories (Fig. 1), indicating a lack of publication bias. However, in category (A) for anthracycline-based chemotherapy for thymoma, two outliers (Fornasiero et al. 1991; Kawashima et al. 2013) from the 95 % tolerance limit were observed. We repeated our analyses excluding these two studies, the response rate in the anthracycline regimen for thymoma was 59.8 %, and the chi-square test still demonstrated a significant difference (*p* < 0.0001).

## Discussion

Our pooled analysis demonstrated that anthracycline-based chemotherapies involving cisplatin appeared to play a key role in improving the response rate of advanced thymoma. In addition, the response rate of advanced thymic carcinoma was significantly higher with cisplatin-based chemotherapy than with carboplatin-based chemotherapy; however, no significant difference in response rates was found between anthracycline-containing regimens and non-anthracycline-containing regimens using platinum-based chemotherapies.

Platinum-based chemotherapy in combination with anthracycline has been suggested as the key regimen for the treatment of advanced thymoma and thymic carcinoma. However, only a small number of patients have been enrolled in phase II trials or retrospective studies regarding the evaluation of therapeutic efficacy. Moreover, until the advent of the 2004 WHO classification, thymoma and thymic carcinoma were not clearly distinguished. However, recent studies have revealed these two carcinomas are different clinical entities in terms of biobehavior and biomarkers (Monica et al. 2013) and clinical outcomes. Therefore, the efficacy of chemotherapy may differ as well; however, the utility of chemotherapy itself remains unclear because none of the trials compared chemotherapy with best supportive care. The present study focused on clarifying the key drugs regarding the treatment of advanced thymic carcinoma. Einhorn’s protocol, which consists of a cisplatin- and anthracycline-based triplet or quartet regimen, such as ADOC (cisplatin, doxorubicin, vincristine and cyclophosphamide) (Fornasiero et al. 1991) or PAC (cisplatin, cyclophosphamide and adriamycin) (Loehrer et al. 1997), is conventionally used for invasive thymoma. The response rates of first-line chemotherapies for thymoma have been compared in phase II trials, and cisplatin and anthracycline have been considered the key drugs. Actually, cisplatin and anthracycline-containing regimens for thymoma have achieved significantly higher response rates than non-anthracycline regimens (Table 1). A dose-dense chemotherapy regimen for thymoma has not always improved efficacy (Kunitoh et al. 2009).

Chemotherapy regimens designed for the treatment of thymoma have also been used for thymic carcinoma. Only two prospective studies have assessed carboplatin and paclitaxel as palliative-intent chemotherapy for advanced stage disease, and one study has assessed induction chemotherapy (Lemma et al. 2011; Park et al. 2013; Takeda et al. 2013). The response rate achieved with carboplatin and paclitaxel was 30–40 %, whereas induction chemotherapy followed by curative-intent treatments involving cisplatin and docetaxel combination chemotherapy achieved a 66.7 % response rate in the subset of patients with thymic carcinoma (Park et al. 2013). Carboplatin-based chemotherapy is broadly used as a combination regimen with less nausea and vomiting or nephrotoxicity than that was reported in the early 2000s. However, more effective anti-emetic agents have recently become available, and consequently, cisplatin-based combination chemotherapy has become more easily available. Therefore, further clinical trials should evaluate cisplatin-based chemotherapy for thymic carcinoma. At present, any platinum-based doublet chemotherapy seems appropriate with the view of maximizing the PFS of patients with thymic carcinoma (Table 2).

The present analysis updated the data regarding the clinical outcome of patients with advanced thymic carcinoma treated using cisplatin and irinotecan, including both squamous cell carcinoma and neuroendocrine carcinoma subtypes, with a response rate of 66.7 %. In the WJTOG 4207L trial, half of the patients were re-diagnosed as having neuroendocrine carcinoma. In thymic squamous cell carcinoma, positivity for biomarkers of neuroendocrine carcinoma, such as synaptophysin, neuron-specific enolase, chromogranin A and CD57, has been previously documented (Lauriola et al. 1998). Cisplatin and irinotecan combination chemotherapy will be one of the choices for treatment as it covers a broad spectrum of subtypes as has been proven to be the case in lung cancer (Noda et al. 2002; Ohe et al. 2007).

The accurate diagnosis of thymoma and thymic carcinoma has recently been discussed as being crucial with regard to the proof of the efficacy of chemotherapy for these rare cancers. Mismatched diagnoses have been suspected in another clinical trial involving rare cancers, the multi-institutional clinical trial regarding imatinib treatment for c-KIT or platelet-derived growth factor receptor (PDGFR)-positive sarcoma (Sugiura et al. 2010). In this trial, the concordance rate between the trial sites and the central review involving immunohistochemical staining was 63.3 %. The results of such studies must be carefully interpreted because a few diagnostic errors in phase II studies with small sample sizes will result in a lack of power to test statistical hypotheses. Investigators who plan clinical trials involving thymic malignancies should incorporate a central review by reliable pathologists who have experience with thymic malignancies. Large-scale regional databases are being established in the USA, Europe and Japan as the first step to curative treatment for thymoma and thymic carcinoma (Detterbeck et al. 2013). This approach appears to be a role model for rare diseases, and the process of establishing databases will help to clarify diagnostic problems. In fact, the WJTOG 4207L trial demonstrated that in 25 % of patients the diagnosis differed between local and central review(Takeda et al. 2013). In thymic malignancies, the reproducibility of pathological diagnosis was examined in an Italian study (Zucali et al. 2013). Clinical trials of rare cancers are limited in that only small numbers of patients can be enrolled. Consequently, misdiagnosis of some patients will invalidate the results of the clinical trial. In common cancers, multi-institutional clinical trials provide a higher level of evidence than trials incorporating a single or a few institutions. However, the opposite is potentially true of clinical trials involving rare cancers. Up to 20 % of thymic malignancies are difficult to diagnose and are termed “borderline.” In fact, diagnostic agreement regarding surgical specimens was low (Zucali et al. 2013). In advanced thymic carcinoma, clinicians should note that the patients are usually diagnosed based on the examination of needle biopsy specimens. As this previous study demonstrated, diagnostic differences occurred among type A thymoma and thymic carcinoma patients (Zucali et al. 2013). Moreover, differentiating between type B thymoma and thymic carcinoma has been difficult for pathologists. In patients with type B3 thymoma, previously called “well-differentiated thymic carcinoma,” the median survival time was 99 months (95 % CI 63.4–134.6), whereas it was 48 months (95 % CI 38.4–94.1; *p*  < 0.001) in patients with thymic carcinoma (Gao et al. 2013). Since the key drugs used for the treatment of thymoma and thymic carcinoma can differ, it is important to have the correct diagnosis to choose the optimal therapy.

The current study had a number of limitations. They included the use of mixed data from prospective and retrospective studies with different criteria, including variations in the precise histological classification of subtypes, staging or assessment criteria. Moreover, detailed patient characteristics could not be completely extracted. However, this is a common limitation in such studies involving small numbers of patients with rare cancers. Furthermore, it was not possible to include time-to-event endpoints, such as progression-free survival and overall survival, in the analysis since individual data regarding chemotherapy for thymomas and thymic carcinomas were not available. It is recommended that the efficacy of chemotherapy be evaluated using time to event, and not only tumor response endpoints (Anderson et al. 1983). Therefore, an optimal chemotherapy regimen for advanced thymomas and thymic carcinomas has still not been established. Medical oncologists should choose the chemotherapy for thymoma and thymic carcinoma based on the treatment setting and/or consideration of the side effects of the chemotherapy. Moreover, there exists a potential publication. For instance, two published studies lay outside the 95 % tolerance limit of the pooled estimate. The reasons for this may be attributed to the following: (1) The study by Fornasiero et al., which was the higher outlier, was the oldest study performed at a single institution. However, prospective studies demonstrated a similar response rate for thymoma (75 and 81.3 %); (2) the study by Kawashima et al. was the only study using amrubicin as anthracycline. Amrubicin is the only totally chemically synthesized anthracycline and commonly inhibits topoisomerase II enzyme; however, the other mechanisms of action for its anticancer activity are speculated to differ as it has weaker DNA intercalation activity than the other anthracycline agents (Noguchi et al. 2005). Therefore, amrubicin may not be the best drug choice for thymic malignancies.

In conclusion, the present pooled analysis demonstrated that platinum with anthracycline-based chemotherapy is an optimal combination for the treatment of advanced thymoma. For advanced thymic carcinoma, cisplatin-based chemotherapy may be superior to carboplatin-based chemotherapy.

## References

[CR1] Agatsuma T, Koizumi T, Kanda S, Ito M, Urushihata K, Yamamoto H, Hanaoka M, Kubo K (2011). Combination chemotherapy with doxorubicin, vincristine, cyclophosphamide, and platinum compounds for advanced thymic carcinoma. J Thorac Oncol.

[CR2] Anderson JR, Cain KC, Gelber RD (1983). Analysis of survival by tumor response. J Clin Oncol.

[CR3] Bernatz PE, Harrison EG, Clagett OT (1961). Thymoma: a clinicopathologic study. J Thorac Cardiovasc Surg.

[CR4] Berruti A, Borasio P, Gerbino A, Gorzegno G, Moschini T, Tampellini M, Ardissone F, Brizzi MP, Dolcetti A, Dogliotti L (1999). Primary chemotherapy with adriamycin, cisplatin, vincristine and cyclophosphamide in locally advanced thymomas: a single institution experience. Br J Cancer.

[CR5] de Jong WK, Blaauwgeers JL, Schaapveld M, Timens W, Klinkenberg TJ, Groen HJ (2008). Thymic epithelial tumours: a population-based study of the incidence, diagnostic procedures and therapy. Eur J Cancer.

[CR6] Detterbeck FC, Asamura H, Crowley J, Falkson C, Giaccone G, Giroux D, Huang J, Kim J, Kondo K, Lucchi M, Marino M, Marom EM, Nicholson A, Okumura M, Ruffini E, van Schil P, Stratton K, Staging, Prognostic Factors C, Members of the Advisory B, Participating Institutions of the Thymic D (2013). The IASLC/ITMIG thymic malignancies staging project: development of a stage classification for thymic malignancies. J Thorac Oncol.

[CR7] Engel P, Marx A, Muller-Hermelink HK (1999). Thymic tumours in Denmark. A retrospective study of 213 cases from 1970-1993. Pathol Res Pract.

[CR8] Engels EA (2010). Epidemiology of thymoma and associated malignancies. J Thorac Oncol.

[CR9] Fornasiero A, Daniele O, Ghiotto C, Piazza M, Fiore-Donati L, Calabro F, Rea F, Fiorentino MV (1991). Chemotherapy for invasive thymoma. A 13-year experience. Cancer.

[CR10] Furugen M, Sekine I, Tsuta K, Horinouchi H, Nokihara H, Yamamoto N, Kubota K, Tamura T (2011). Combination chemotherapy with carboplatin and paclitaxel for advanced thymic cancer. Jpn J Clin Oncol.

[CR11] Gao L, Wang C, Fang W, Zhang J, Lv C, Fu S (2013). Outcome of multimodality treatment for 188 cases of type b3 thymoma. J Thorac Oncol.

[CR12] Giaccone G, Ardizzoni A, Kirkpatrick A, Clerico M, Sahmoud T, van Zandwijk N (1996). Cisplatin and etoposide combination chemotherapy for locally advanced or metastatic thymoma. A phase II study of the European Organization for Research and Treatment of Cancer Lung Cancer Cooperative Group. J Clin Oncol.

[CR13] Girard N, Lal R, Wakelee H, Riely GJ, Loehrer PJ (2011). Chemotherapy definitions and policies for thymic malignancies. J Thorac Oncol.

[CR14] Grassin F, Paleiron N, Andre M, Caliandro R, Bretel JJ, Terrier P, Margery J, Le Chevalier T, Ruffie P (2011). Combined etoposide, ifosfamide, and cisplatin in the treatment of patients with advanced thymoma and thymic carcinoma. A French experience. J Thorac Oncol.

[CR15] Huang J, Detterbeck FC, Wang Z, Loehrer PJ (2010). Standard outcome measures for thymic malignancies. J Thorac Oncol.

[CR16] Igawa S, Murakami H, Takahashi T, Nakamura Y, Tsuya A, Naito T, Kaira K, Ono A, Shukuya T, Tamiya A, Endo M, Yamamoto N (2010). Efficacy of chemotherapy with carboplatin and paclitaxel for unresectable thymic carcinoma. Lung Cancer.

[CR17] Kawashima Y, Inoue A, Sugawara S, Harada M, Kobayashi K, Kozuki T, Kuyama S, Sakakibara T, Maemondo M, Asahina H, Hisamoto A, Nakagawa T, Nukiwa T (2013) Phase II study of amrubicin (AMR) and carboplatin (CBDCA) for invasive thymoma (IT) and thymic carcinoma (TC): NJLCG0803. ASCO Meeting Abstracts 31(15_suppl):7530

[CR18] Kim ES, Putnam JB, Komaki R, Walsh GL, Ro JY, Shin HJ, Truong M, Moon H, Swisher SG, Fossella FV, Khuri FR, Hong WK, Shin DM (2004). Phase II study of a multidisciplinary approach with induction chemotherapy, followed by surgical resection, radiation therapy, and consolidation chemotherapy for unresectable malignant thymomas: final report. Lung Cancer.

[CR19] Kunitoh H, Tamura T, Shibata T, Nakagawa K, Takeda K, Nishiwaki Y, Osaki Y, Noda K, Yokoyama A, Saijo N, Jcog Lung Cancer Study Group TJ (2009). A phase-II trial of dose-dense chemotherapy in patients with disseminated thymoma: report of a Japan Clinical Oncology Group trial (JCOG 9605). Br J Cancer.

[CR20] Lauriola L, Erlandson RA, Rosai J (1998). Neuroendocrine differentiation is a common feature of thymic carcinoma. Am J Surg Pathol.

[CR21] Lemma GL, Lee JW, Aisner SC, Langer CJ, Tester WJ, Johnson DH, Loehrer PJ (2011). Phase II study of carboplatin and paclitaxel in advanced thymoma and thymic carcinoma. J Clin Oncol.

[CR22] Levine GD, Rosai J (1978). Thymic hyperplasia and neoplasia: a review of current concepts. Hum Pathol.

[CR23] Loehrer PJ Sr, Kim K, Aisner SC, Livingston R, Einhorn LH, Johnson D, Blum R (1994) Cisplatin plus doxorubicin plus cyclophosphamide in metastatic or recurrent thymoma: final results of an intergroup trial. The Eastern Cooperative Oncology Group, Southwest Oncology Group, and Southeastern Cancer Study Group. J Clin Oncol 12(6):1164–1168 10.1200/JCO.1994.12.6.1164 8201378

[CR24] Loehrer PJ, Chen M, Kim K, Aisner SC, Einhorn LH, Livingston R, Johnson D (1997). Cisplatin, doxorubicin, and cyclophosphamide plus thoracic radiation therapy for limited-stage unresectable thymoma: an intergroup trial. J Clin Oncol.

[CR25] Loehrer PJ, Jiroutek M, Aisner S, Aisner J, Green M, Thomas CR, Livingston R, Johnson DH (2001). Combined etoposide, ifosfamide, and cisplatin in the treatment of patients with advanced thymoma and thymic carcinoma: an intergroup trial. Cancer.

[CR26] Lucchi M, Melfi F, Dini P, Basolo F, Viti A, Givigliano F, Angeletti CA, Mussi A (2006). Neoadjuvant chemotherapy for stage III and IVA thymomas: a single-institution experience with a long follow-up. J Thorac Oncol.

[CR27] Masaoka A (2010). Staging system of thymoma. J Thorac Oncol.

[CR28] Monica V, Familiari U, Chiusa L, Rossi G, Novero D, Busso S, Ruffini E, Ardissone F, Scagliotti GV, Papotti M (2013). Messenger RNA and protein expression of thymidylate synthase and DNA repair genes in thymic tumors. Lung Cancer.

[CR29] Muller-Hermelink HK, Marino M, Palestro G (1986). Pathology of thymic epithelial tumors. Curr Top Pathol.

[CR30] Noda K, Nishiwaki Y, Kawahara M, Negoro S, Sugiura T, Yokoyama A, Fukuoka M, Mori K, Watanabe K, Tamura T, Yamamoto S, Saijo N (2002). Irinotecan plus cisplatin compared with etoposide plus cisplatin for extensive small-cell lung cancer. N Engl J Med.

[CR31] Noguchi T, Hanada M, Yamaoka T (2005) Development of a novel anti-tumor drug ‘amrubicin’, a completely synthetic anthracycline. SUMITOMO KAGAKU 2005-II:1–11

[CR32] Ohe Y, Ohashi Y, Kubota K, Tamura T, Nakagawa K, Negoro S, Nishiwaki Y, Saijo N, Ariyoshi Y, Fukuoka M (2007). Randomized phase III study of cisplatin plus irinotecan versus carboplatin plus paclitaxel, cisplatin plus gemcitabine, and cisplatin plus vinorelbine for advanced non-small-cell lung cancer: Four-Arm Cooperative Study in Japan. Ann Oncol.

[CR33] Okuma Y, Hosomi Y, Takagi Y, Iguchi M, Okamura T, Shibuya M (2011). Cisplatin and irinotecan combination chemotherapy for advanced thymic carcinoma: evaluation of efficacy and toxicity. Lung Cancer.

[CR34] Park S, Ahn MJ, Ahn JS, Sun JM, Shim YM, Kim J, Choi YS, Kim K, Shin S, Ahn Y, Kwon OJ, Kim H, Lee SJ, Chang WJ, Park K (2013). A prospective phase II trial of induction chemotherapy with docetaxel/cisplatin for Masaoka stage III/IV thymic epithelial tumors. J Thorac Oncol.

[CR35] Rea F, Sartori F, Loy M, Calabro F, Fornasiero A, Daniele O, Altavilla G (1993). Chemotherapy and operation for invasive thymoma. J Thorac Cardiovasc Surg.

[CR36] Rosai J (1999) Histological typing of tumours of the thymus. In: World Health Organization International Histological Classification of Tumours. Springer, Berlin

[CR37] Schmitt J, Loehrer PJ (2010). The role of chemotherapy in advanced thymoma. J Thorac Oncol.

[CR38] Sugiura H, Fujiwara Y, Ando M, Kawai A, Ogose A, Ozaki T, Yokoyama R, Hiruma T, Ishii T, Morioka H, Mugishima H (2010). Multicenter phase II trial assessing effectiveness of imatinib mesylate on relapsed or refractory KIT-positive or PDGFR-positive sarcoma. J Orthop Sci.

[CR39] Takeda K, Hirai F, Yamanaka T, Taguchi K, Daga H, Shimizu J, Kogure Y, Kimura T, Tanaka K, Iwamoto Y, Ono A, Sasaki H, Fukuoka J, Nishiyama K, Seto T, Ichinose Y, Nakagawa K, Nakanishi Y, West Japan Oncology Group (2013) A multicenter prospective study of carboplatin and paclitaxel for advanced thymic carcinoma: West Japan Oncology Group 4207L. ASCO Meeting Abstracts 31(15_suppl):7529

[CR40] Travis W, Brambilla W, Müller-Hermelink H, Harris C (2004) World Health Organization classification of tumors. Pathology and genetics of tumors of the lung, pleura, thymus and heart. Chapter 3. IARC press, Lyon

[CR41] Weksler B, Dhupar R, Parikh V, Nason KS, Pennathur A, Ferson PF (2013). Thymic carcinoma: a multivariate analysis of factors predictive of survival in 290 patients. Ann Thorac Surg.

[CR42] Yoh K, Goto K, Ishii G, Niho S, Ohmatsu H, Kubota K, Kakinuma R, Nagai K, Suga M, Nishiwaki Y (2003). Weekly chemotherapy with cisplatin, vincristine, doxorubicin, and etoposide is an effective treatment for advanced thymic carcinoma. Cancer.

[CR43] Yokoi K, Matsuguma H, Nakahara R, Kondo T, Kamiyama Y, Mori K, Miyazawa N (2007). Multidisciplinary treatment for advanced invasive thymoma with cisplatin, doxorubicin, and methylprednisolone. J Thorac Oncol.

[CR44] Zucali PA, Di Tommaso L, Petrini I, Battista S, Lee HS, Merino M, Lorenzi E, Voulaz E, De Vincenzo F, Simonelli M, Roncalli M, Giordano L, Alloisio M, Santoro A, Giaccone G (2013). Reproducibility of the WHO classification of thymomas: practical implications. Lung Cancer.

